# Harvard

**Published:** 1897-09

**Authors:** 


### HARVARD UNIVERSITY.

The vacancy in the Governing Board for 1900 has been filled by the selection of George Frisbie Hoar, LL.D., Worcester. Dr. Ogden is one of the recent instructors in chemistry. One of the unusual features at Harvard is that in the course the clinical exercises are given by different instructors, thus doing away with the serious objections arising from the instruction being given by one man, however good he may be.

